# Single-nucleus RNA sequencing reveals ARHGAP28 expression of podocytes as a biomarker in human diabetic nephropathy

**DOI:** 10.1515/med-2025-1146

**Published:** 2025-04-02

**Authors:** Fengxia Zhang, Xianhu Tang, Zhimei Zeng, Chunyu Cao, Caocui Yun, Yue Shen, Chaohong Nie, Ying Xiong, Mao Chulian, Yueheng Wu, Ruiquan Xu

**Affiliations:** Department of Nephrology, First Affiliated Hospital of Gannan Medical University, Ganzhou, Jiangxi, China; Department of Stomatology, First Affiliated Hospital of Gannan Medical University, Ganzhou, Jiangxi, China; Department of Nephrology, Shaoxing People’s Hospital, Shaoxing, Zhejiang, China; Guangdong Cardiovascular Institute, Guangdong Provincial People’s Hospital, Guangdong Academy of Medical Sciences, Guangzhou, Guangdong, China; Department of Urology, First Affiliated Hospital of Gannan Medical University, Ganzhou, 341000, Jiangxi, China

**Keywords:** diabetic kidney disease, DKD, single-nucleus RNA sequencing, snRNA-seq, podocyte, ARHGAP28

## Abstract

**Introduction:**

Diabetic kidney disease (DKD) represents serious diabetes-associated complications, and podocyte loss is an important histologic sign of DKD. The cellular and molecular profiles of podocytes in DKD have yet to be fully elucidated.

**Methods:**

This study analyzed kidney-related single-nucleus RNA-seq datasets (GSE131882, GSE121862, and GSE141115) and human diabetic kidney glomeruli transcriptome profiling (GSE30122). ARHGAP28 expression was validated by western blot and immunohistochemistry.

**Results:**

In human kidney tissues, 154 differentially expressed genes (DEGs) were identified in podocytes, which were enriched in biological processes related to nephron development and extracellular matrix–receptor interactions. Similarly, in the mouse kidney, 344 DEGs were found, clustering in pathways associated with renal development and signaling mechanisms like PI3K/Akt (phosphatidylinositol-3 kinase/protein kinase B) and PPAR (peroxisome proliferator-activated receptor). In diabetic human kidney glomeruli, 438 DEGs were identified, showing significant enrichment in pathways related to diabetic nephropathy. Venn analysis revealed 22 DEGs common across human and mouse podocytes and diabetic glomeruli, with ARHGAP28 being notably overexpressed in podocytes. The diabetic nephropathy model using db/db mice showed that ARHGAP28 expression was significantly upregulated in the kidney cortex and glomeruli. *In vitro* studies using a high-glucose podocyte model corroborated these findings.

**Conclusions:**

Collectively, this study provides an insight into the function and diagnosis of DKD and indicates that ARHGAP28 in podocytes is a potential biomarker of DKD.

## 1 Introduction

Globally, diabetes is the main reason for renal failure. Complications of the kidney microvascular are common in diabetic patients, leading to proteinuria [1–4], which induced ∼35% of the diabetic cases progressing into diabetic nephropathy (diabetic kidney disease [DKD]) [5–8]. Pathological features of DKD include glomerular basement membrane thickening, mesangial expansion, and podocyte loss, which ultimately lead to end-stage renal disease [9,10]. Podocyte injury is an essential factor leading to the occurrence and progression of DKD [11–15]. Because of the complexity of metabolic disorders, the treatment will be more difficult than other kidney diseases once diabetes has developed into DKD. Therefore, understanding the molecular mechanism of renal podocytes is crucial for the clinical treatment of DKD.

Although RNA sequencing has been used in several studies of DKD, relatively little is known about pathways and cell types that contribute to DKD progression. Single-nucleus RNA sequencing (snRNA-seq) is a powerful tool for deciphering the cellular landscape for complex tissues, which can significantly advance the characterization of cell-type diversity and composition and advance our knowledge in biological systems [16,17]. For example, Wilson et al. revealed the single-cell transcriptomic landscape of early human DKD [18]. Nevertheless, more specific analysis and research for underlying genetic mechanism of early DKD remain rare. Deciphering single-cell atlas in DKD provides a valuable data resource for illustrating DKD from multiple perspectives and opens up the possibility of developing new and more targeted treatment methods for DKD.

This investigation aimed to verify the vital role of podocytes and potential biomarkers in DKD based on kidney-related single-nucleus RNA-seq datasets (GSE131882, GSE121862, and GSE141115), human diabetic kidney glomeruli transcriptome profiling (GSE30122), and integrated bioinformatics analysis. The snRNA-seq datasets of human DKD were downloaded from a public database. The essential differentially expressed genes (DEGs) in podocytes were identified, and functional enrichment analysis was performed to dig out the core signaling pathways. In addition, the potential genetic biomarker in DKD was also identified and verified using a *db/db* mice model and a podocyte model. The identification of podocytes and potential biomarker offers the possibility for preventing or substantially delaying the onset of DKD.

## 2 Materials and methods

### 2.1 Data acquisition

The scRNA-seq datasets of DKD samples included in this study for analysis (Accession numbers GSE131882 [18], GSE121862 [19], GSE141115 [20], and GSE30122) were downloaded from NCBI’s Gene Expression Omnibus (GEO, http://www.ncbi.nlm.nih.gov/geo/). GSE131882 included three early human DKD samples and three control samples. GSE121862 included two kidney regions (cortex and medulla) obtained from 15 different individuals. GSE141115 comprised 12 single nuclei using healthy adult mouse kidneys, with 3 biological replicates included per condition. Transcriptome profiling by an array of GSE30122 contained 19 DKD samples and 50 corresponding controls, and the glomerulus samples were used for analysis in this study.

### 2.2 Cell clustering and DEGs

The R software packages Seurat (4.3.0) and clusterProfiler (4.6.0) were used to process data. We filtered out the genes expressed in less than three cells and cells with less than 500 genes. The data were for single-nuclear sequencing, so mitochondrial gene filtering was not involved. Then, the top 2,000 hypervariable genes (HVGS) were calculated by normalized (log-normalized) and scaled and analyzed by integration with canonical correlation analysis. First, principal component analysis (PCA) was run, and the first 30 principal components (PCs) were selected with a resolution of 0.5 for clustering. The cell type annotation was based on a typical marker. We calculated the threshold of marker gene with “min.pct” = 0.25 and “log FC threshold” = 0.25 (FC = fold change). The differential genes were identified with a threshold *P* value <0.05. Subgroup cells were extracted from the total cells, and the PCA and clustering were recalculated. The first five PCs were used for clustering with a resolution of 0.2.

### 2.3 Differential expression analysis

The differentially expressed mRNAs in podocytes were identified based on expression profiling. Quality control, normalization, differential analysis, and enrichment analysis of the original data were carried out using the Affy algorithm, with the criterion |log2(FC)| >2 and *P* < 0.05.

### 2.4 Functional enrichment analysis

ClusterProfile in R was utilized to conduct gene ontology (GO) and Kyoto Encyclopedia of Genes and Genomes (KEGG) analyses [21]. *P*-value < 0.05 was set as the cut-off criterion. Gene set enrichment analysis (GSEA) was performed using GSEA software [22]. The H (hallmark) gene sets were downloaded from the molecular signatures database (MSigDB) (https://www.gsea-msigdb.org/gsea/msigdb/), and gene sets were rated by the gene set variation analysis (GSVA) method and using the GSEABase algorithm.

### 2.5 Clinical samples

Renal biopsy specimens from patients diagnosed with type 2 diabetic nephropathy were collected from the First Affiliated Hospital of Gannan Medical University. The diagnosis of diabetic nephropathy was confirmed for all specimens based on biopsy results. Additionally, adjacent normal tissue specimens were obtained from patients undergoing renal cancer resection at the same institution. All collected specimens were processed and preserved in paraffin for subsequent analyses. This study was approved by the Ethics Committee of the First Affiliated Hospital of Gannan Medical University. Written informed consent was obtained from all participants prior to specimen collection.

### 2.6 Animal model

Eight-week-old C57BLKS/J *db/db* diabetic (*n* = 6) and *db/m* (*n* = 6) normal male mice were obtained from the Model Animal Research Center of Nanjing University (Nanjing, China). All animals were maintained in a temperature-controlled room at the animal center of South China Agricultural University Institutional Animal Care. All experimental protocols were performed in accordance with the Ethics Review Committee for Animal Experimentation of South China Agricultural University (No. 2020D077). After 24 weeks, the serum samples (extracted from eyeballs), 24 h urine samples, and kidney tissues were collected for further study.

### 2.7 Periodic acid-Shiff (PAS) staining

PAS staining was used to detect glycogen and other polysaccharide substances. Briefly, the renal tissue was collected and soaked in 10% neutral-buffered formalin (NBF). The kidney tissue was embedded in wax and sectioned, and then it was dewaxed with water to prepare paraffin sections (4 μm). After being washed with double-distilled water (ddH_2_O) for 2 min, the slices were dripped with a solution of PA and oxidized for 5 min. Slices were instilled with the Schiff reagent and infected for 15 min. Then, hematoxylin staining solution was added dropwise to stain the nucleus for 2 min. The stained sections were washed with running water for 5 min and then differentiated with an acidic ethanol differentiation solution. Next, the slices were washed with Scott’s reverse blue water for 3 min. The final slices were sealed with neutral gum.

### 2.8 Immunohistochemistry

The kidney tissue was fixed in 4% paraformaldehyde for 24 h. The embedded paraffin blocks were sliced and dewaxed. Then, immunohistochemical staining was performed. The sodium citrate antigen repair solution was added to the sample and fixed with microwaves for 30 min, allowing it to cool naturally at room temperature. A solution of 3% H_2_O_2_ and 5% BSA blocking solution were dripped onto a glass slide and incubated at room temperature and at 37°C in the dark for 30 min. The sealing liquid was gently shaken, and the prepared first antibody ARHGAP28 (1:100; rabbit, 84642; Novus) was added to the slide and incubated for 2 days in a wet box at 4°C. The biotin-labeled goat anti-rabbit IgG (No. KIT-9710; Servicebio) was added, and the slides were placed in an incubator and incubated at 37°C for 30 min. 3,3′-Diaminobenzidine chromogenic solution was added to stain the specimen until a brown color is visible under a microscope. The sample was re-stained with hematoxylin for 5 min, then stained with PBS for 5 min until the blue color returned, immersed in anhydrous ethanol for 10 min, and washed with xylene for 10 min. Finally, the sample was dried and sealed with neutral gum.

### 2.9 Podocyte culture

Human glomerular podocytes were cultured in Dulbecco’s modified Eagle medium high glucose complete medium in type-I collagen coated flasks at 33°C in RPMI-1640 supplemented with 10% fetal bovine serum and 50 U/mL γ-interferon (IFN-γ). To induce differentiation, podocytes were cultured at 37°C in the absence of IFN-γ for 10–14 days. Podocytes were divided into two groups: HG and NG groups. The HG group was incubated with 30.0 mmol/L glucose  and NG group with 5.3 mmol/L glucose for 48 h. After treatment, podocytes were collected for mRNA or protein extraction.

### 2.10 Quantitative reverse-transcription polymerase chain reaction (qRT-PCR)

RNA in cells or tissues was extracted with Trizol. The concentration and purity of RNA were determined with NanoDrop 2000C. RNA were reverse-transcribed into cDNA using the PCR kit. Then, the qRT-PCR was conducted according to the following settings: 95°C for 35 s, 60°C for 30 s, 95°C for 10 s, and 65°C for 5 s. After the reaction, the amplification curve and the fusion curve were confirmed, and the 2-△△CT (CT = threshold cycle) value representing the relative expression of mRNA was calculated. The sequences synthesized by Fuzhou Shangya Biosynthesis are as follows:

GAPDH-F: 5′-TGTGTCCGTCGTGGA TCTGA-3′,

GAPDH-R: 5′-TTGCTGTTGAAGTCGCAGGAG-3′

ARHGAP28-F: 5′-TTAGTCGCTCCAACTCTCAAGC-3′,

ARHGAP28-R: 5′-GATACTCTCAATTTCCCGCAGG-3′.

### 2.11 Western blot analysis

Two groups of podocytes with different glucose concentrations were lysed with RIPA lysis buffer. Protein concentrations were quantified using the Bradford assay kit (Beyotime). They were separated by 9% sodium dodecyl sulfate-polyacrylamide gel electrophoresis with 30 μg protein. Then, they were transferred to the polyvinylidene fluoride (PVDF) membrane (Amersham Biosciences, CA, USA). After blocking, the membrane was incubated overnight with the ARHGAP28 antibody (1:100; rabbit, 84642; Novus) at 4°C. After washing, Goat anti-rabbit IgG (1:10,000, Jackson ImmunoResearch Labs, West grove, PA, USA) bound with horseradish peroxidase was added, and the samples were cultured at room temperature for 1 h. PVDF films were washed and developed with ECL Plus Western blotting reagent (Pluslight, Forever Lighting, China) and then exposed to X-ray films (Kodak, Rochester, NY, USA). The developed strips were quantified by BandScan software.

### 2.12 Statistical analysis

Statistical analyses were conducted using GraphPad Prism 7.0 (GraphPad, La Jolla, CA, USA). Unpaired *t* tests were used to compare the mean values between groups. All results are presented as mean ± standard deviation (SD). *P* < 0.05 was considered to be statistically significant.


**Informed consent:** The patients/participants provided their written informed consent to participate in this study. Written informed consent was obtained from the individual(s) for the publication of any potentially identifiable images or data included in this article.
**Ethical approval:** The animal study protocol was approved by the Ethics Review Committee for Animal Experimentation of First Affiliated Hospital of Gannan Medical University (No. 2020D077). The collection of clinical samples was approved by the Ethics Committee of First Affiliated Hospital of Gannan Medical University (No. 2024168).

## 3 Results

### 3.1 Single-nucleus analysis reveals DEGs of podocytes in the adult human kidney

**Figure 1:**
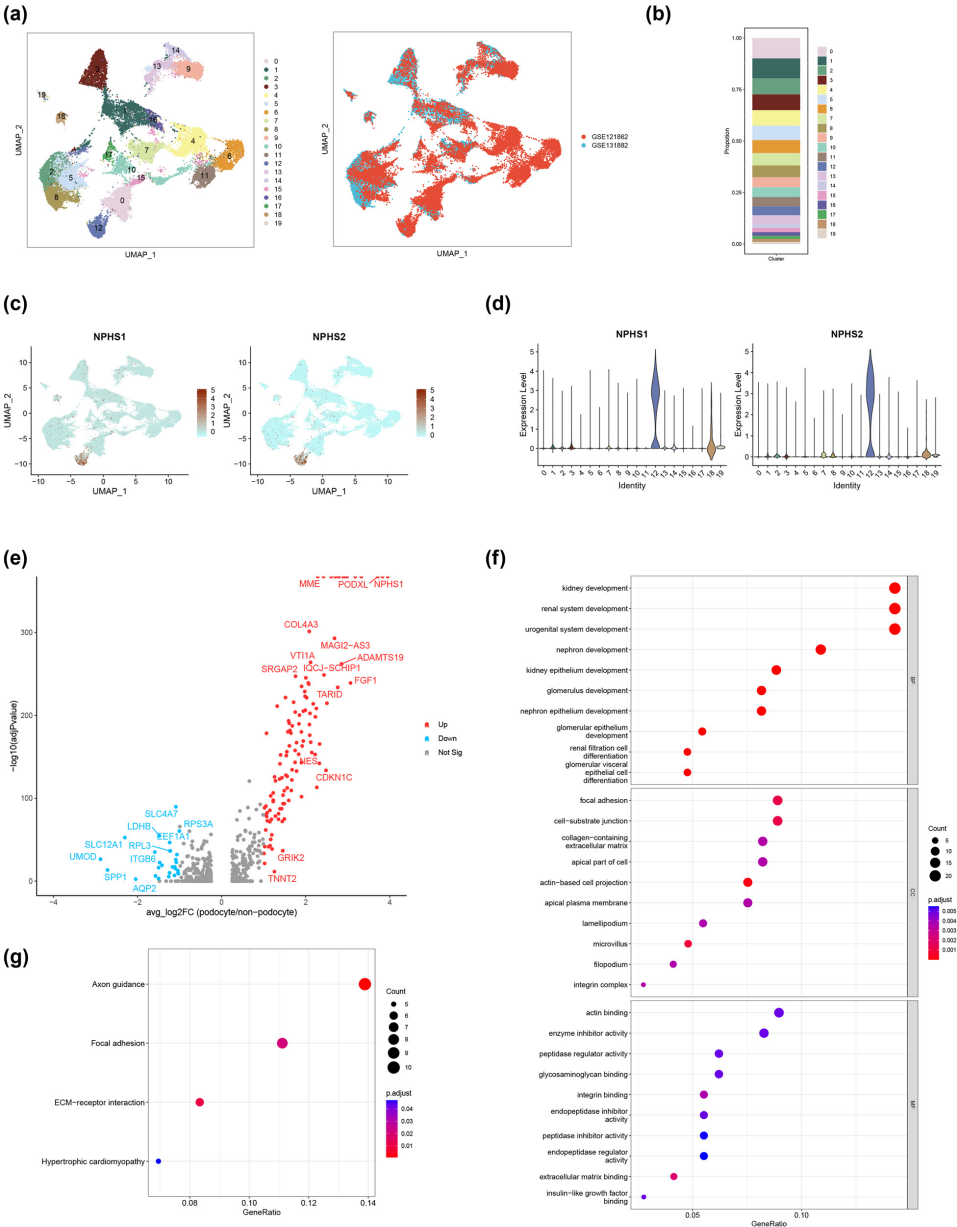
Single-nucleus analysis of the adult human kidney (GSE121862 and GSE131882) and DEGs of podocytes. (a) Cell clusters of adult human kidney and source of the datasets. (b) Cell composition of adult human kidney. (c) Expression of podocyte markers (feature plot). (d) Expression of podocyte markers (violin plot). (e) Volcano plot of DEGs (podocytes vs non-podocytes). (f) GO enrichment of podocyte DEGs (top ten terms). (g) KEGG enrichment of podocyte DEGs.

The single-nucleus expression profiling of the adult human kidney (GSE131882 and GSE121862) was downloaded from the GEO database to perform the bioinformatics analysis. As shown in Figure 1a, a total of 20 cell clusters in human kidney tissue were identified and presented in the uniform manifold approximation and projection (UMAP) method. The cell composition of the 20 clusters in the adult human kidney is presented in Figure 1b. Focusing on the expression level of markers of podocytes, cluster 12 was identified as the podocyte in the adult kidney (Figure 1c and d). Furthermore, the DEGs between the podocyte and non-podocyte types were screened out. As shown in Figure 1e, 154 DEGs were identified, which included the 126 upregulated and 28 downregulated DEGs (screening criteria: *P* < 0.05 and |avg_log2FC| >1). To further investigate the function of these DEGs, enrichment analysis was performed. The enrichment of GO terms indicated that these DEGs were clustered in the biological process associated with kidney and nephron development (Figure 1f). The enrichment of KEGG pathways revealed that DEGs participated in the extracellular matrix (ECM)–receptor interaction (Figure 1g), which is also related to chronic kidney disease [23]. These results reveal that DEGs of podocytes participate in nephron development in the adult human kidney.

### 3.2 Single-nucleus analysis reveals DEGs of podocytes in the adult mouse kidney

**Figure 2:**
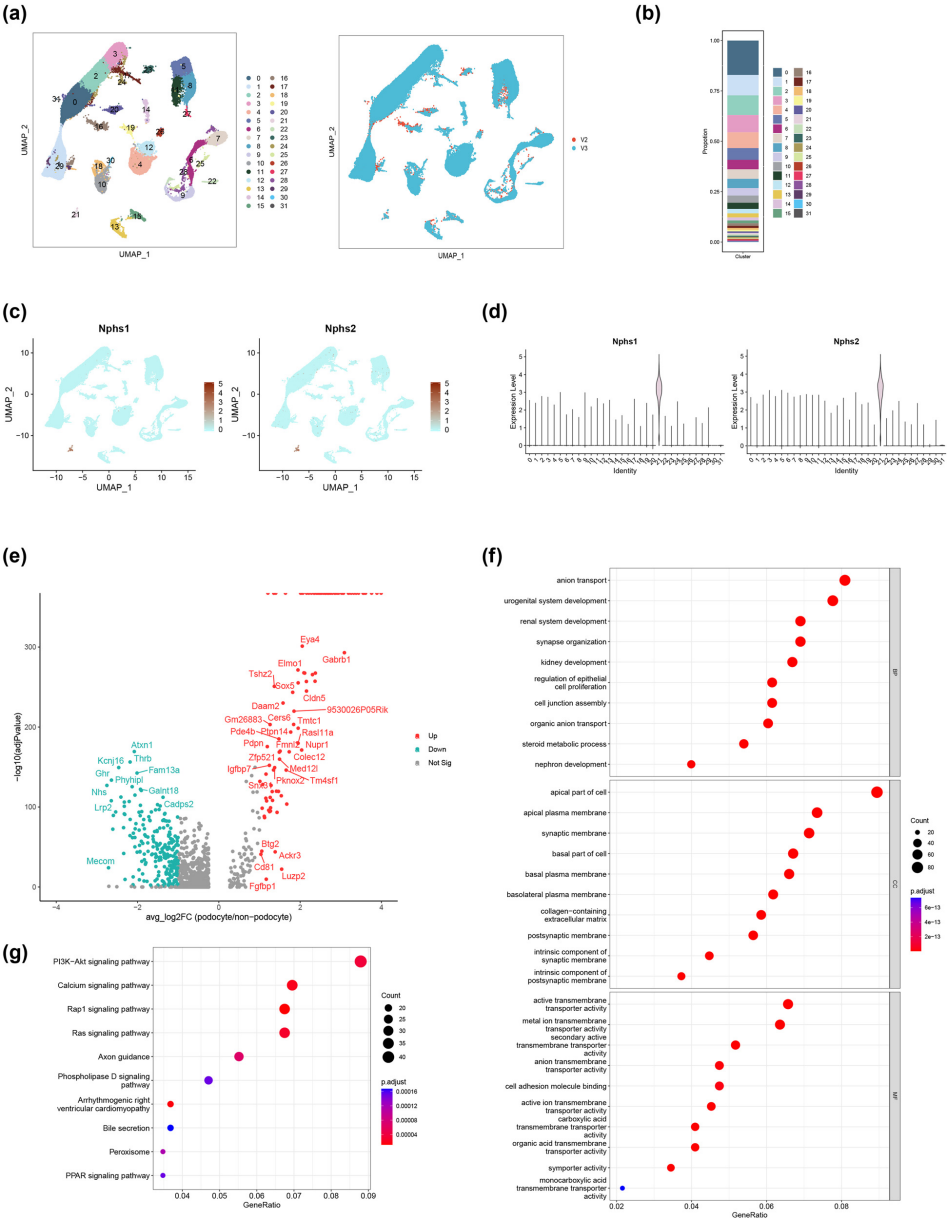
Single-nucleus analysis of adult mouse kidney (GSE141115) and DEGs of podocytes. (a) Cell clusters of adult mouse kidney and sequencing protocol of the datasets. (b) Cell composition of adult mouse kidney. (c) Expression of podocyte markers (feature plot). (d) Expression of podocyte markers (violin plot). (e) Volcano plot of DEGs (podocytes vs non-podocytes). (f) GO enrichment of podocyte DEGs (top ten terms). (g) KEGG enrichment of podocyte DEGs (top 10 terms).

In the next step, we subsequently explored the single-nucleus expression profiling in the adult mouse kidney. Based on the single-nucleus datasets of GSE141115, a total of 32 clusters were identified in the adult mouse kidney (Figure 2a). The cell composition of adult mouse kidney is presented in Figure 2b. According to the expression signatures of podocyte markers, cluster 21 was identified to be the podocyte (Figure 2c and d). To screen out characteristic genes of podocytes at the molecular level, the DEGs in-between the podocyte and non-podocyte types were identified. As shown in Figure 2e, 122 upregulated and 222 downregulated DEGs were screened out (screening criteria: *P* < 0.05 and |avg_log2FC| >1). These DEGs were clustered in GO terms for renal system, kidney, and nephron development (Figure 2f). In addition, these DEGs were enriched in PI3K/Akt (phosphatidylinositol-3 kinase/protein kinase B), calcium, Rap1 (Ras-related protein 1), and PPAR (peroxisome proliferator-activated receptor) signaling pathways based on KEGG enrichment analysis (Figure 2g). The results of single-nucleus analysis reveal that DEGs of podocytes participate in nephron development in the adult mouse kidney.

### 3.3 Identification of DEGs in human diabetic kidney glomeruli

**Figure 3:**
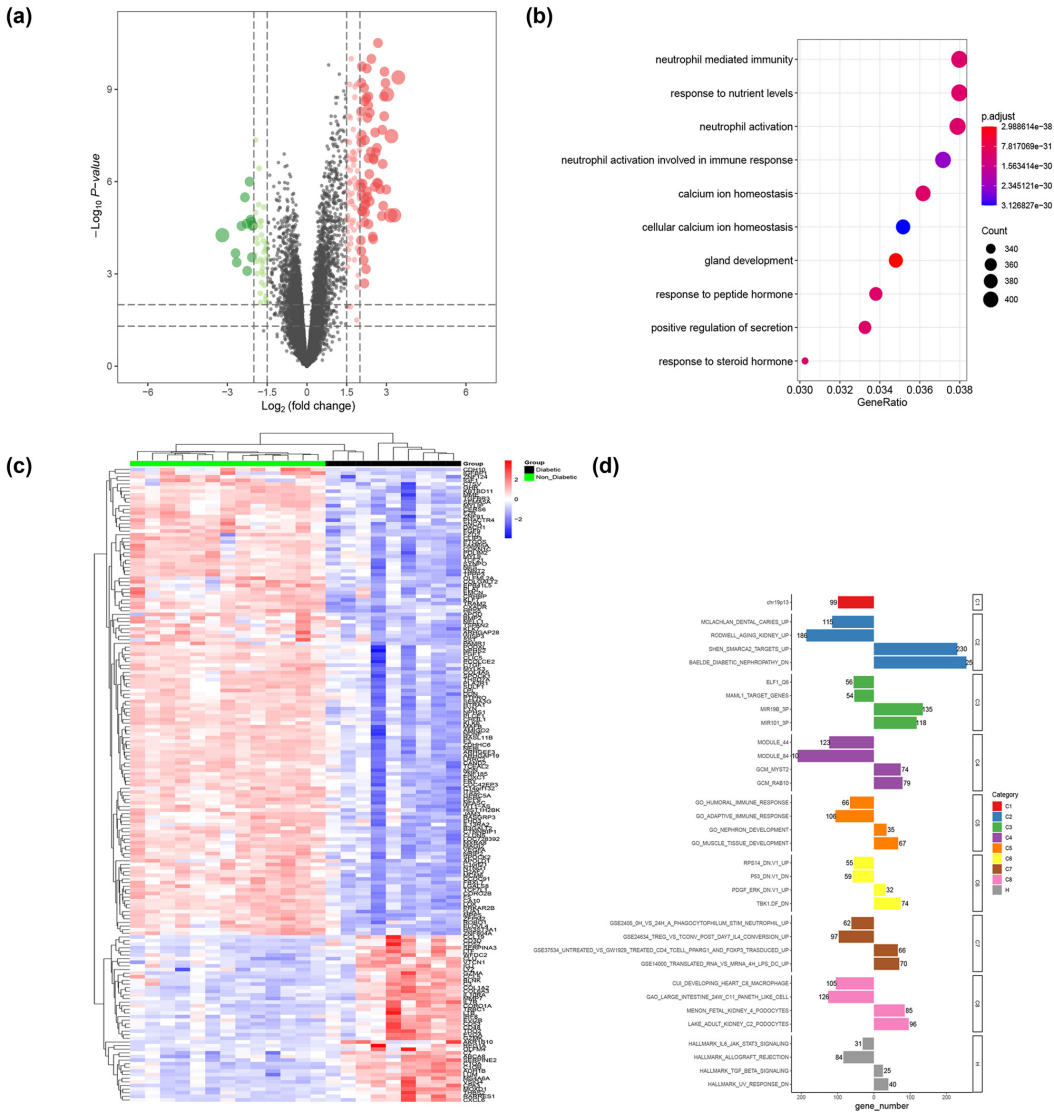
DEGs of diabetic human kidney glomeruli microarray dataset (GSE30122). (a) Volcano plot of DEGs. (b) GO BP enrichment of DEGs. (c) Heatmap of DEG expression. (d) GSEA enrichment of DEGs.

Glomerular podocytes are essential in maintaining the glomerular filtration barrier function [24]. Hence, the diabetic human kidney glomeruli microarray dataset (GSE30122) was downloaded for further analysis. As shown in Figure 3a, the volcano plot demonstrated that 323 upregulated DEGs and 115 downregulated DEGs in-between the diabetic human kidney and control human kidney samples were identified (screening criteria: *P* < 0.05 and |avg_log2FC| >1). Clustering relationships between samples or genes are displayed in a heat map (Figure 3c). The DEGs were used to conduct the GO enrichment, and the biological process of neutrophil was significantly enriched (Figure 3b). Hyperglycemia can affect the adhesion, chemotaxis, phagocytosis, and bactericidal effects of neutrophils [25]. Furthermore, the GSEA enrichment of DEGs was also conducted, and diabetic nephropathy associated pathway was significantly activated (Figure 3d). These results illustrate the vital role of DEGs in diabetic human kidney glomeruli to participate in diabetic nephropathy.

### 3.4 ARHGAP28 is highly expressed in podocytes in single-cell data of kidney

**Figure 4:**
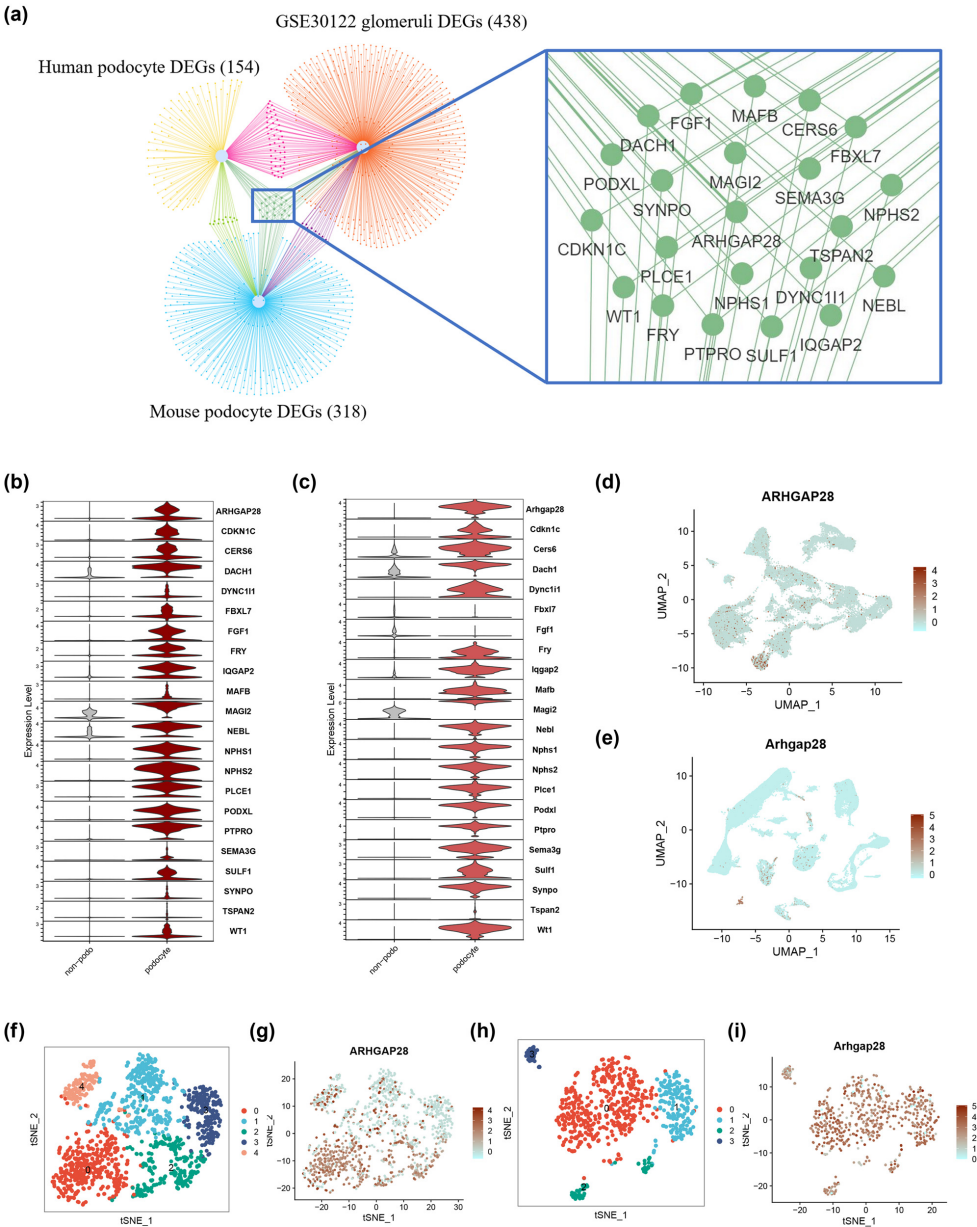
Narrow down target genes and ARHGAP28 expression in single-cell data. (a) Overlap between DEGs of human podocytes, human orthologs of DEGs of mouse podocytes, and diabetic kidney glomeruli DEGs. (b) Expression of 22 overlap genes in human single-cell data (violin plot); all cells are grouped into podocytes and non-podocytes. (c) Expression of 22 overlap genes in mouse single-cell data (violin plot); all cells are grouped into podocytes and non-podocytes. (d) Expression of ARHGP28 in human single cell data (feature plot). (e) Expression of ARHGAP28 in mouse single cell data (feature plot). (f) Sub-clusters of human podocytes. (g) Expression of ARHGAP28 in human podocyte sub-clusters (feature plot). (h) Sub-clusters of mouse podocytes. (i) Expression of ARHGAP28 in mouse podocyte sub-clusters (feature plot).

To find out the vital DEGs that are commonly expressed in human podocytes, human orthologs of DEGs of mouse podocytes, and diabetic kidney glomeruli DEGs, Venn analysis was performed. As illustrated in Figure 4a, the overlap of 3 parts of DEGs were presented, and 22 common DEGs were identified, including ARHGAP28, MAGI2, NPHS1, SYNPO, TSPAN2, and so on. Expression of 22 overlapping common DEGs in human single-cell datasets is shown in a violin plot, and all cells were grouped into podocytes and non-podocytes (Figure 4b and c). Based on the expression level of the 22 overlapping common DEGs in podocytes and non-podocytes, ARHGAP28 was found to be remarkably highly expressed in podocytes. The expression level of ARHGAP28 was obviously presented in cluster 12 (podocyte) in the UMAP of human single-cell data (Figure 4d). Besides, the expression of ARHGAP28 in mouse single-cell data was obviously observed in cluster 21 (podocytes) (Figure 4e). The sub-clusters of human podocytes (Figure 4f) and expression of ARHGAP28 in human podocyte sub-clusters (feature plot) (Figure 4g) were shown. The sub-clusters of human podocytes (Figure 4h) and expression of ARHGAP28 in mouse podocyte sub-clusters (feature plot) (Figure 4i) were also shown. The above proofs reveal that ARHGAP28 is highly expressed in podocytes in single-cell data of human diabetic kidney.

### 3.5 ARHGAP28 is highly expressed in the DKD animal model and the podocyte model

**Figure 5:**
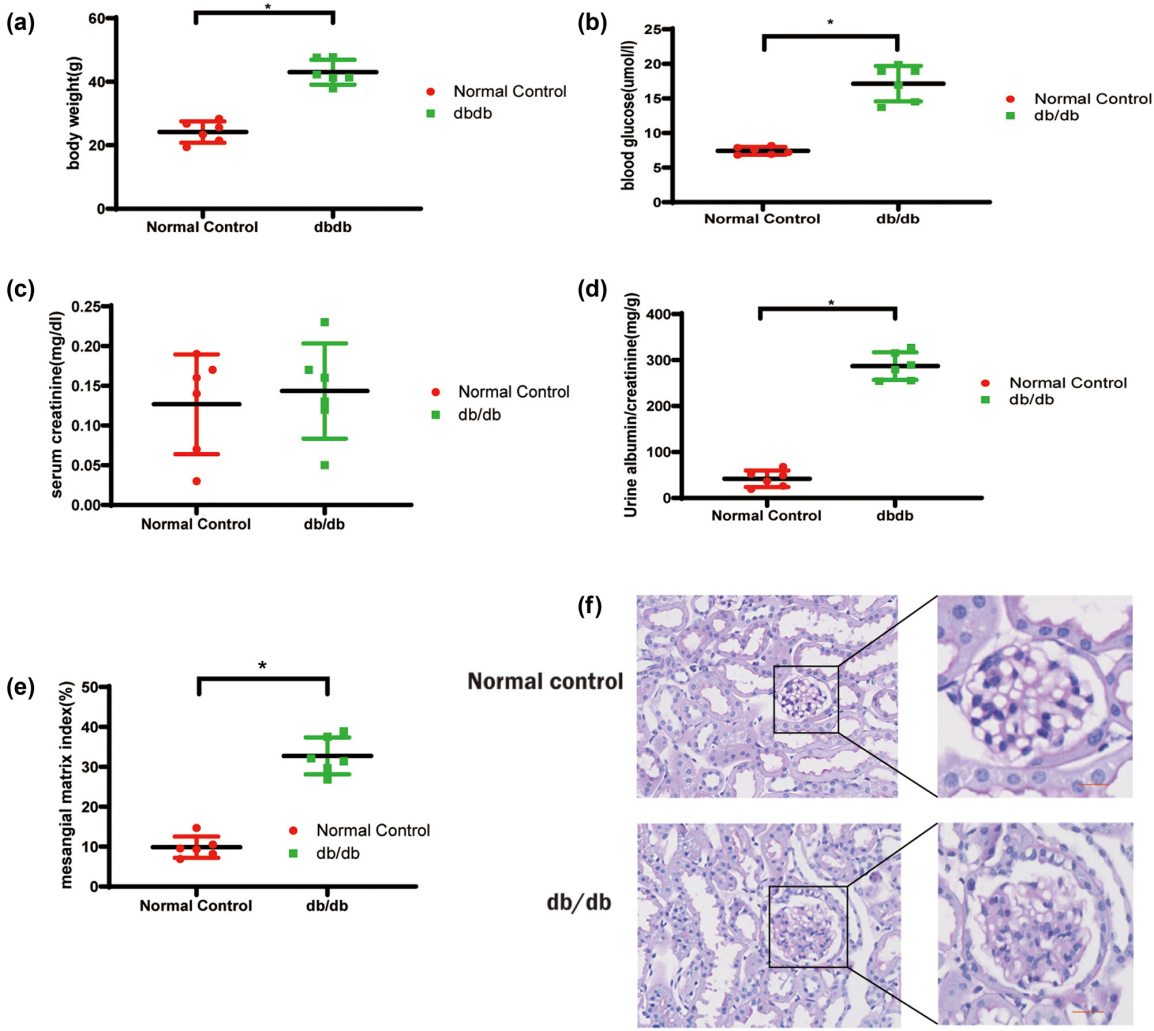
DKD modeling. (a) Body weight, (b) blood glucose, (c) serum creatinine, (d) urine albumin/creatinine, (e) semi-quantitative analysis of mesangial matrix, (f) representative photomicrographs of PAS staining of glomeruli of db/db mice and nondiabetic mice. *P < 0.05. Scale bar = 25 µm.

Further, in order to verify the expression level of ARHGAP28 in DKD, we used *db/db* diabetic mice to construct a DKD animal model. As shown in Figure 5a, the body weight is enhanced in *db/db* diabetic mice compared with controls. In addition, a significantly increased concentration of biomarkers of renal injury (blood glucose and urine albumin/creatinine) was observed in the *db/db* diabetic mice model (Figure 5b and d). Besides, obvious glomerular injury was observed in histopathological examination, such as glomerular hyaline droplets and increased mesangial matrix (Figure 5e and f). These results implied the successful construction of the *db/db* diabetic mice model.

**Figure 6:**
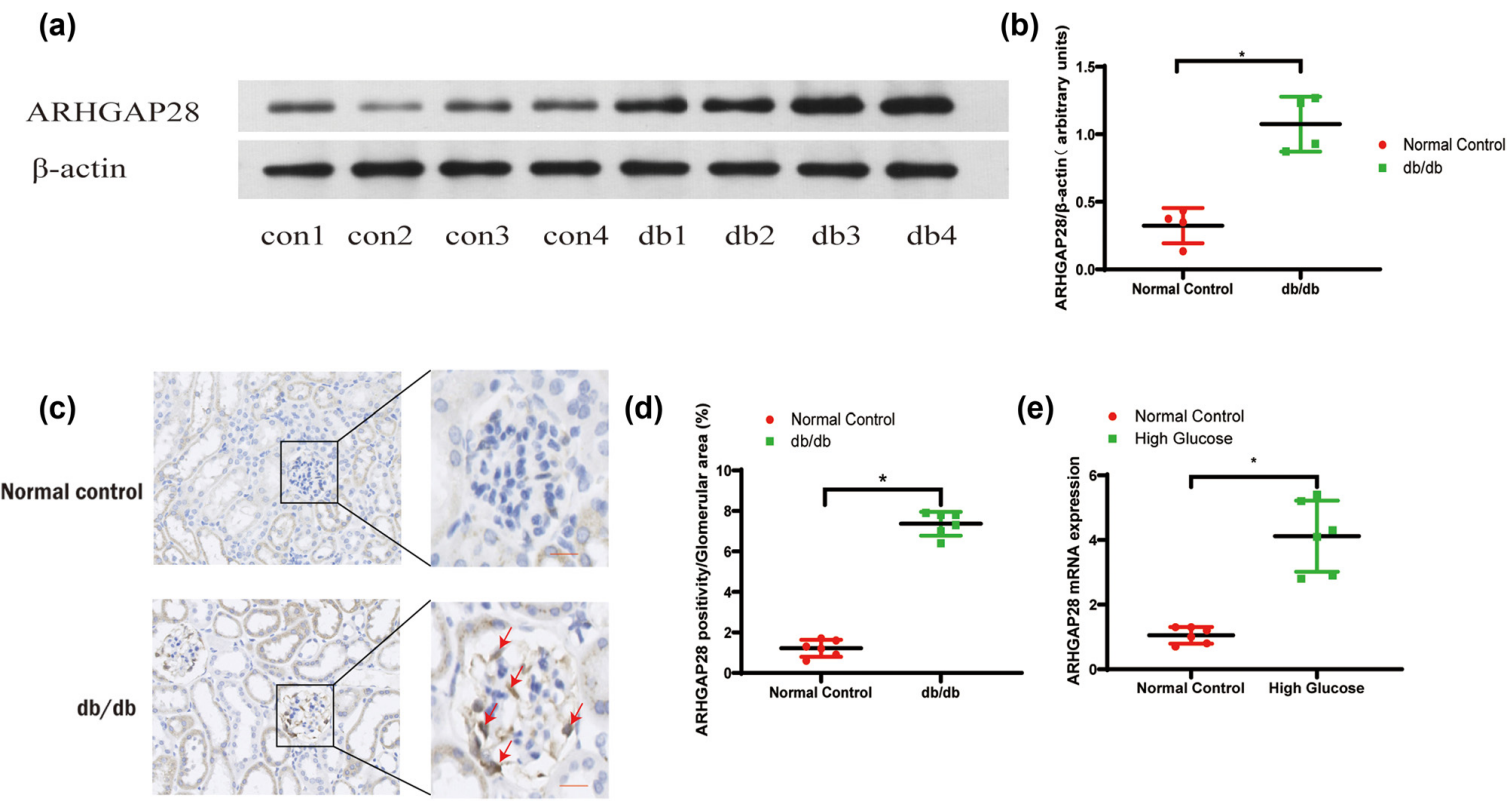
Validation of ARHGAP28 in DKD. (a) Western blot gel showing ARHGAP28 protein expression in the mouse kidney cortex. (b) Semiquantitative analysis of ARHGAP28 protein expression in the mouse kidney cortex. (c) Images of glomerular ARHGAP28 immunohistochemical staining from mouse kidney sections. (d) Semi-quantitative analysis of ARHGAP28 immunohistochemical staining from mouse kidney sections. *P < 0.05. Scale bar = 25 µm. Red arrows indicate the positive staining of ARHGAP28. (e) Podocytes were cultured *in vitro* and stimulated by high glucose. Expression of mRNA of ARHGAP28 in the treated group and normal control group was tested by qPCR. *P < 0.05.

Western blot analysis showed that ARHGAP28 protein was significantly upregulated in the mouse kidney cortex in the db/db group compared to the normal group (Figure 6a and b). The immunohistochemical staining of glomerular ARHGAP28 from mouse kidney sections is presented in Figure 6c, and significantly higher protein levels of ARHGAP28 were detected in *db/db* mouse kidney tissues compared to normal tissues (Figure 6d). Consistently, ARHGAP28 protein expression was higher in the human kidney tissues from DKD patients than that from controls (Figure S1). In *in vitro* studies, we established a DKD cellular model by treating the podocytes with high glucose, and we consistently found that ARHGAP28 expression in podocytes stimulated by high glucose was higher than that in controls (Figure 6e). These results indicate that ARHGAP28 was a highly expressed gene and a potential biomarker in DKD *in vitro* and *in vivo*.

## 4 Discussion

DKD is a serious diabetic complication and a major cause of end-stage renal disease [26]. Histologic signs of diabetic nephropathy include glomerular basement membrane thickening, mesangial expansion, and podocyte loss, resulting in subsequent end-stage renal disease [27]. To date, DKD is characterized by damage to both the glomerulus and tubulointerstitium [28]. However, the accompanying gene changes in specific cells that precede overt DKD is poorly understood. Newer genetic markers for early-stage DKD are urgently needed. The snRNA-seq is a precise tool to reveal complex and rare cell populations and track distinct cell lineage trajectories in development [29,30]. This method has attracted the researchers’ attention and increasingly used in disease studies [31]. Here, analysis based on snRNA-seq data sets was performed to further explore the central remodeling cell subtypes and potential biomarkers in DKD.

Podocytes are an important cell type in DKD. Glomerular podocytes are an essential glomerular filtration barrier. As severe podocyte injury results in proteinuria in patients with DKD, the investigation of genetic biomarkers in podocytes provides a deeper understanding for novel diagnosis and treatment [32]. Structural changes or injuries in podocytes are related to renal injury leading to severe renal insufficiency and DKD [33]. In addition, Mahtal et al. recently revealed the intricate relation of endothelial cells with podocytes. During DKD, endothelial cells and podocytes are stressed and damaged [1]. In this study, we identified podocytes as significant cell type in DKD.

Podocyte loss is an important histologic sign of DKD [34]. However, the signaling pathways of podocyte loss contributing to DKD progression are not fully understood. Our investigation identified DEGs in podocytes. Enrichment analysis of DEGs indicated that DEGs in podocytes may be involved in kidney/nephron development. The GSEA results indicate that the diabetic nephropathy associated pathway was significantly activated when the DEGs of podocytes were upregulated. Hence, we focused on the potential contribution of DEGs in podocytes to single-cell data of kidneys. Single-nucleus RNA sequencing of both human and mouse kidneys revealed that ARHGAP28 is among the top DEGs in podocytes, with its expression particularly enriched in processes linked to nephron development and ECM–receptor interactions – pathways crucial for maintaining kidney structure and function. Further analysis of diabetic human kidney glomeruli demonstrated that ARHGAP28 is also highly upregulated in diabetic conditions, suggesting its potential role in the pathogenesis of diabetic nephropathy. Venn analysis identified ARHGAP28 as one of the 22 DEGs common to human and mouse podocytes and diabetic kidney glomeruli, highlighting its conserved function across species and disease models. Given its consistent overexpression in both normal and diabetic conditions, ARHGAP28 emerges as a prime candidate for functional assays to explore its role in podocyte biology and its potential as a therapeutic target in kidney diseases.

ARHGAP28 is a critical Rho GTPase activating protein that regulates the formation and migration of the cytoskeleton by converting the active state of RhoA into an inactive state [35]. This protein is mainly located in the cytoplasm and is highly expressed in various cell types, including podocytes in the kidney, hepatic stellate cells in the liver, outer root sheath cells in the skin, and sperm cells in the testes. Research has found that the promoter region of ARHGAP28 exhibits significant methylation in colon cancer cells with high metastatic potential. This change may lead to the persistent activation of RhoA, thereby affecting the metastatic potential of cancer cells [36]. Additionally, ARHGAP28 is also associated with the recurrence and progression of breast cancer [37]. In the development of meningiomas, ARHGAP28 plays an important role, particularly in pathways related to cell migration and invasiveness [38,39]. Its variation may cause vasoconstriction and spasms in cerebral blood vessels, leading to migraine and potentially exacerbated inflammatory responses in the brain [40]. Although ARHGAP28 has significant impacts on various physiological and disease processes, there are currently no reports on its role in the development of DKD, especially concerning podocyte injury. This phenomenon highlights the importance and urgency of further exploring the relationship between ARHGAP28 and DKD. ARHGAP28 is important in Rho-mediated cell adhesion establishment [41]. Yeung et al. revealed that ARHGAP28 is a RhoGAP that inactivates RhoA and downregulates stress fibers [36]. Stress fibers are contractile actomyosin bundles and are associated with actin cables that span along the foot processes [42]. Rho family are modulators of actin cytoskeletal dynamics [43]. Podocyte actin cytoskeleton is involved in kidney podocyte functions [44,45]. Saito et al. indicated that afadin modulates RhoA/Rho-associated protein kinase signaling to affect actin stress fibers in kidney podocytes [46]. These pieces of evidence may suggest that ARHGAP28 is of great importance in podocytes. In our results, the expression of ARHGAP28 in podocytes was elevated compared to controls. Biochemical analysis and immunochemical results confirmed this result. For the first time, we identified ARHGAP28 as a potential genetic biomarker of DKD.

## 5 Conclusions

In summary, we revealed podocytes as the essential cell subtype, which plays an essential role in the regulation of DKD based on single-nucleus-sequencing analysis and bioinformatics analysis. Podocytes are involved in kidney and nephron development. ARHGAP28 is a potential biomarker of podocytes involved in the pathogenesis of DKD. This study provides an insight into the disease progression or signaling pathways amenable to intervention in DKD. Further clinical validation to explore the underlying mechanism of ARHGAP28 in podocytes of DKD is needed.

## Supplementary Material

Supplementary Figure

## References

[1] Mahtal N, Lenoir O, Tharaux P. Glomerular endothelial cell crosstalk with podocytes in diabetic kidney disease. Front Med. 2021;8:659013. 10.3389/fmed.2021.659013 PMC8024520 33842514

[2] Jha R, Lopez-Trevino S, Kankanamalage HR, Jha JC. Diabetes and renal complications: An overview on pathophysiology, biomarkers and therapeutic interventions. Biomedicines. 2024;12(5):1098. 10.3390/biomedicines12051098 PMC11118045 38791060

[3] Fang Z, Liu R, Xie J, He JC. Molecular mechanism of renal lipid accumulation in diabetic kidney disease. J Cell Mol Med. 2024;28(11):e18364. 10.1111/jcmm.18364 PMC11151220 38837668

[4] Deng L, Shi C, Li R, Zhang Y, Wangc X, Cai G, et al. The mechanisms underlying Chinese medicines to treat inflammation in diabetic kidney disease. J Ethnopharmacol. 2024;7:118424. 10.1016/j.jep.2024.118424 38844252

[5] Ruiz-Ortega M, Rodrigues-Diez RR, Lavoz C, Rayego-Mateos S. Special issue “Diabetic nephropathy: Diagnosis, prevention and treatment”. J Clin Med. 2020;9(3):813. 10.3390/jcm9030813 PMC7141346 32192024

[6] Nakagawa T, Tanabe K, Croker BP, Johnson RJ, Grant MB, Kosugi T, et al. Endothelial dysfunction as a potential contributor in diabetic nephropathy. Nat Rev Nephrol. 2011;7(1):36–44. 10.1038/nrneph.2010.152 PMC3653134 21045790

[7] Soltani-Fard E, Taghvimi S, Karimi F, Vahedi F, Khatami SH, Behrooj H, et al. Urinary biomarkers in diabetic nephropathy. Clin Chim Acta. 2024;561:119762. 10.1016/j.cca.2024.119762 38844018

[8] Cheng G, Liu Y, Guo R, Wang H, Zhang W, Wang Y. Molecular mechanisms of gut microbiota in diabetic nephropathy. Diabetes Res Clin Pract. 2024;213:111726. 10.1016/j.diabres.2024.111726 38844054

[9] Qi C, Mao X, Zhang Z, Wu H. Classification and differential diagnosis of diabetic nephropathy. J Diabetes Res. 2017;2017:8637138. 10.1155/2017/8637138 PMC5337846 28316995

[10] Singh DK, Winocour P, Farrington K. Oxidative stress in early diabetic nephropathy: fueling the fire. Nat Rev Endocrinol. 2011;7(3):176–84. 10.1038/nrendo.2010.212 21151200

[11] Tung C, Hsu Y, Shih Y, Chang P, Lin C. Glomerular mesangial cell and podocyte injuries in diabetic nephropathy. Nephrology (Carlton, Vic). 2018;32–7. 10.1111/nep.13451 30298646

[12] Dai H, Liu Q, Liu B. Research progress on mechanism of podocyte depletion in diabetic nephropathy. J Diabetes Res. 2017;2017:2615286. 10.1155/2017/2615286 PMC5534294 28791309

[13] Barutta F, Bellini S, Gruden G. Mechanisms of podocyte injury and implications for diabetic nephropathy. Clin Sci (London, England: 1979). 2022;136(7):493–520. 10.1042/CS20210625 PMC9008595 35415751

[14] Li X, Gao L, Li X, Xia J, Pan Y, Bai C. Autophagy, pyroptosis and ferroptosis are rising stars in the pathogenesis of diabetic nephropathy. Diabetes Metab Syndr Obes. 2024;17:1289–99. 10.2147/DMSO.S450695 PMC10949337 38505538

[15] Gong L, Wang R, Wang X, Liu J, Han Z, Li Q, et al. Research progress of natural active compounds on improving podocyte function to reduce proteinuria in diabetic kidney disease. Ren Fail. 2023;45(2):2290930. 10.1080/0886022X.2023.2290930 PMC11001328 38073545

[16] Ding J, Adiconis X, Simmons S, Kowalczyk M, Hession C, Marjanovic N, et al. Systematic comparison of single-cell and single-nucleus RNA-sequencing methods. Nat Biotechnol. 2020;38(6):737–46. 10.1038/s41587-020-0465-8 PMC7289686 32341560

[17] Alvarez M, Benhammou J, Darci-Maher N, French S, Han S, Sinsheimer J, et al. Human liver single nucleus and single cell RNA sequencing identify a hepatocellular carcinoma-associated cell-type affecting survival. Genome Med. 2022;14(1):50. 10.1186/s13073-022-01055-5 PMC9115949 35581624

[18] Wilson P, Wu H, Kirita Y, Uchimura K, Ledru N, Rennke H, et al. The single-cell transcriptomic landscape of early human diabetic nephropathy. Proc Natl Acad Sci U S A. 2019;116(39):19619–25. 10.1073/pnas.1908706116 PMC6765272 31506348

[19] Lake B, Chen S, Hoshi M, Plongthongkum N, Salamon D, Knoten A, et al. A single-nucleus RNA-sequencing pipeline to decipher the molecular anatomy and pathophysiology of human kidneys. Nat Commun. 2019;10(1):2832. 10.1038/s41467-019-10861-2 PMC6597610 31249312

[20] Denisenko E, Guo B, Jones M, Hou R, de Kock L, Lassmann T, et al. Systematic assessment of tissue dissociation and storage biases in single-cell and single-nucleus RNA-seq workflows. Genome Biol. 2020;21(1):130. 10.1186/s13059-020-02048-6 PMC7265231 32487174

[21] Yu G, Wang LG, Han Y, He QY. clusterProfiler: An R package for comparing biological themes among gene clusters. Omics. 2012;16(5):284–7. 10.1089/omi.2011.0118 PMC3339379 22455463

[22] Subramanian A, Tamayo P, Mootha VK, Mukherjee S, Ebert BL, Gillette MA, et al. Gene set enrichment analysis: A knowledge-based approach for interpreting genome-wide expression profiles. Proc Natl Acad Sci U S A. 2005;102(43):15545–50. 10.1073/pnas.0506580102 PMC1239896 16199517

[23] Yu W, Li Y, Wang Z, Liu L, Liu J, Ding F, et al. Transcriptomic changes in human renal proximal tubular cells revealed under hypoxic conditions by RNA sequencing. Int J Mol Med. 2016;38(3):894–902. 10.3892/ijmm.2016.2677 27432315

[24] Nagata M. Podocyte injury and its consequences. Kidney Int. 2016;89(6):1221–30. 10.1016/j.kint.2016.01.012 27165817

[25] Fainsod-Levi T, Gershkovitz M, Völs S, Kumar S, Khawaled S, Sagiv J, et al. Hyperglycemia impairs neutrophil mobilization leading to enhanced metastatic seeding. Cell Rep. 2017;21(9):2384–92. 10.1016/j.celrep.2017.11.010 29186678

[26] Colhoun HM, Marcovecchio ML. Biomarkers of diabetic kidney disease. Diabetologia. Clinical and Experimental Diabetes and Metabolism = Organ of the European Association for the Study of Diabetes (EASD). 2018;61:996–1011. 10.1007/s00125-018-4567-5 PMC6448994 29520581

[27] Lin J, Susztak K. Podocytes: The weakest link in diabetic kidney disease? Curr Diabetes Rep. 2016;16(5):45. 10.1007/s11892-016-0735-5 PMC5064850 27053072

[28] Ioannou K. Diabetic nephropathy: Is it always there? Assumptions, weaknesses and pitfalls in the diagnosis. Hormones (Athens). 2017;16(4):351–61. 10.14310/horm.2002.1755 29518755

[29] Zhu Y, Huang Y, Tan Y, Zhao W, Tian Q. Single-cell RNA sequencing in hematological diseases. Proteomics. 2020;20:e1900228. 10.1002/pmic.201900228 32181578

[30] Hwang B, Lee J, Bang D. Author correction: Single-cell RNA sequencing technologies and bioinformatics pipelines. Exp Mol Med. 2021;53(5):1005. 10.1038/s12276-021-00615-w PMC8178331 34045654

[31] Hedlund E, Deng Q. Single-cell RNA sequencing: Technical advancements and biological applications. Mol Asp Med. 2018;59:36–46. 10.1016/j.mam.2017.07.003 28754496

[32] Yasuda-Yamahara M, Kume S, Tagawa A, Maegawa H, Uzu T. Emerging role of podocyte autophagy in the progression of diabetic nephropathy. Autophagy. 2015;11(12):2385–6. 10.1080/15548627.2015.1115173 PMC4835189 26565953

[33] Tung C-W, Hsu Y-C, Shih Y-H, Chang P-J, Lin C-L. Glomerular mesangial cell and podocyte injuries in diabetic nephropathy. Nephrology (Carlton). 2018;23(Suppl 4):32–7. 10.1111/nep.13451 30298646

[34] Zhu W, Li Y, Zeng H, Liu X, Sun Y, Jiang L, et al. Carnosine alleviates podocyte injury in diabetic nephropathy by targeting caspase-1-mediated pyroptosis. Int Immunopharmacol. 2021;101:108236. 10.1016/j.intimp.2021.108236 34653727

[35] Kasuya K, Nagakawa Y, Hosokawa Y, Sahara Y, Takishita C, Nakajima T, et al. RhoA activity increases due to hypermethylation of ARHGAP28 in a highly liver-metastatic colon cancer cell line. Biomed Rep. 2016;4(3):335–9. 10.3892/br.2016.582 PMC4774340 26998271

[36] Yeung CY, Taylor SH, Garva R, Holmes DF, Zeef LA, Soininen R, et al. Arhgap28 is a RhoGAP that inactivates RhoA and downregulates stress fibers. PLoS One. 2014;9(9):e107036. 10.1371/journal.pone.0107036 PMC4161385 25211221

[37] Jeon S, Sung H, Choi J-Y, Song M, Park SK, Yoo K-Y, et al. Abstract 1656: Genome wide identification of susceptibility loci in breast cancer survival. Cancer Res. 2012;72(8_Suppl):1656.

[38] Schulten HJ, Hussein D. Array expression meta-analysis of cancer stem cell genes identifies upregulation of PODXL especially in DCC low expression meningiomas. PLoS One. 2019;14(5):e0215452. 10.1371/journal.pone.0215452 PMC6513070 31083655

[39] Fèvre-Montange M, Champier J, Durand A, Wierinckx A, Honnorat J, Guyotat J, et al. Microarray gene expression profiling in meningiomas: Differential expression according to grade or histopathological subtype. Int J Oncol. 2009;35(6):1395–407. 10.3892/ijo_00000457 19885562

[40] Jiang Y, Wu R, Chen C, You ZF, Luo X, Wang XP. Six novel rare non-synonymous mutations for migraine without aura identified by exome sequencing. J Neurogenet. 2015;29(4):188–94. 10.3109/01677063.2015.1122787 26814133

[41] Wang L, Li Y, Lyu Y, Wen H, Feng C. Association between copy-number alteration of + 20q, -14q and -18p and cross-sensitivity to tyrosine kinase inhibitors in clear-cell renal cell carcinoma. Cancer Cell Int. 2020;20:482. 10.1186/s12935-020-01585-1 PMC7541266 33041663

[42] Pellegrin S, Mellor H. Actin stress fibres. J Cell Sci. 2007;120(Pt 20):3491–9. 10.1242/jcs.018473 17928305

[43] Kistler A, Altintas M, Reiser J. Podocyte GTPases regulate kidney filter dynamics. Kidney Int. 2012;81(11):1053–5. 10.1038/ki.2012.12 PMC3354621 22584591

[44] Ichimura K, Kurihara H, Sakai T. Actin filament organization of foot processes in vertebrate glomerular podocytes. Cell Tissue Res. 2007;329(3):541–57. 10.1007/s00441-007-0440-4 17605050

[45] Ichimura K, Kurihara H, Sakai T. Actin filament organization of foot processes in rat podocytes. J Histochem Cytochem: Off J Histochem Soc. 2003;51(12):1589–600. 10.1177/002215540305101203 14623927

[46] Saito K, Shiino T, Kurihara H, Harita Y, Hattori S, Ohta Y. Afadin regulates RhoA/Rho-associated protein kinase signaling to control formation of actin stress fibers in kidney podocytes. Cytoskeleton (Hoboken, NJ). 2015;72(3):146–56. 10.1002/cm.21211 25712270

