# Supplementary material for: Single-nucleus RNA sequencing reveals ARHGAP28 expression of podocytes as a biomarker in human diabetic nephropathy
Source: Open Med (Wars). 2025 Apr 2;20(1):20251146. doi: 10.1515/med-2025-1146 (PMC11967489; doi:10.1515/med-2025-1146)
Supplement: Supplementary Figure [file med-2025-1146-sm.pdf]

Supplementary material

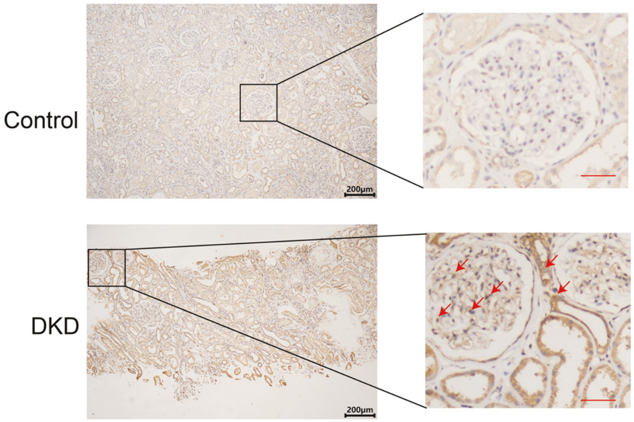

Figure S1: Expression of ARHGAP28 in human kidney tissues. Images of ARHGAP28 immunohistochemistry from human kidney sections. Scale bar = 25 μm. Red arrows indicate the positive staining of ARHGAP28.
